# Metabolites and Genes behind Cardiac Metabolic Remodeling in Mice with Type 1 Diabetes Mellitus

**DOI:** 10.3390/ijms23031392

**Published:** 2022-01-26

**Authors:** Tyler N. Kambis, Hamid R. Shahshahan, Paras K. Mishra

**Affiliations:** Department of Cellular and Integrative Physiology, University of Nebraska Medical Center, Omaha, NE 68198, USA; tyler.kambis@unmc.edu (T.N.K.); hamid.shahshahan@unmc.edu (H.R.S.)

**Keywords:** beta-oxidation, diabetic cardiomyopathy, fatty acid, genomics, ketogenesis, ketolysis, metabolomics, next generation sequencing, TCA cycle

## Abstract

Metabolic remodeling is at the heart of diabetic cardiomyopathy. High glycemic fluctuations increase metabolic stress in the type 1 diabetes mellitus (T1DM) heart. There is a lack of understanding on how metabolites and genes affect metabolic remodeling in the T1DM heart. We hypothesize that differential expression of metabolic genes and metabolites synergistically influence metabolic remodeling preceding T1DM cardiomyopathy. To test our hypothesis, we conducted high throughput analysis of hearts from adult male hyperglycemic *Ins2*^+/−^ (Akita) and littermate normoglycemic *Ins2*^+/+^ (WT) mice. The Akita mouse is a spontaneous, genetic model of T1DM that develops increased levels of consistent glycemic variability without the off-target cardiotoxic effects present in chemically- induced models of T1DM. After validating the presence of a T1DM phenotype, we conducted metabolomics via LC-MS analysis and genomics via next-generation sequencing in left ventricle tissue from the Akita heart. Ingenuity Pathway Analyses revealed that 108 and 30 metabolic pathways were disrupted within the metabolomics and genomics datasets, respectively. Notably, a comparison between the two analyses showed 15 commonly disrupted pathways, including ketogenesis, ketolysis, cholesterol biosynthesis, acetyl CoA hydrolysis, and fatty acid biosynthesis and beta-oxidation. These identified metabolic pathways predicted by the differential expression of metabolites and genes provide the foundation for understanding metabolic remodeling in the T1DM heart. By limited experiment, we revealed a predicted disruption in the metabolites and genes behind T1DM cardiac metabolic derangement. Future studies targeting these genes and metabolites will unravel novel therapies to prevent/improve metabolic remodeling in the T1DM heart.

## 1. Introduction

The limited impact of intensive glycemic control on reducing the risk of heart failure in diabetes mellitus (DM) patients, combined with the complex etiology, and rapidly increasing prevalence of DM make diabetic cardiomyopathy (DMCM) a significant clinical problem with dire consequences [1,2,3]. A loss of metabolic flexibility due to the decreased substrate utilization of glucose causes metabolic stress and instigates metabolic remodeling in DMCM [4]. Glycemic control further increases metabolic adaptive stress due to inconsistent fluctuations in glucose uptake, particularly in the T1DM heart. Metabolic adaptations occur so cardiomyocytes can maintain a consistent turnover of ATP. These adaptations precede and increase the risk of cardiovascular events in T1DM patients [5]. Due to DMCM’s complex etiology, there is a lack of knowledge on how differentially expressed genes and metabolites can be used to understand the metabolic remodeling behind DMCM.

The *Ins2*^+/−^ Akita mouse model spontaneously develops T1DM due to a mutation in the *Insulin 2* (*Ins2*) gene, which is orthologous to human *Insulin* gene. This induces pancreatic beta cell destruction, subsequently demonstrating the major downstream features of T1DM [6]. This model is similar to T1DM in human in that as both develop a mutation in the gene that encodes insulin [7,8]. The Akita mouse is considered a better model compared to streptozotocin or alloxan-induced T1DM, which demonstrate diabetes-independent cardiotoxic and genotoxic effects [9,10]. Furthermore, blood glucose levels in Akita mice maintain a relatively constant increase in hyperglycemia post four weeks of age, reaching an average of 500 mg/dL at eight weeks of age. This consistency in hyperglycemia is difficult to achieve in chemically-induced T1DM, where blood glucose levels vary between normo- and hyper-glycemia [11]. Therefore, we utilized the T1DM Akita model for our subsequent studies.

Our previous publications revealed increased levels of intramyocardial lipid accumulation in the Akita heart [12]. This results from adaption in metabolic substrate use due to T1DM-induced cardiac glucose deprivation. As fatty acid becomes the preferred substrate, myocardial lipid accumulation increases due to oversaturated beta-oxidation. This causes lipotoxicity preceding DMCM in the Akita heart [13]. While studies on cardiac metabolic remodeling are focused on T2DM, the metabolic status of the T1DM and T2DM hearts are different [14,15]. Comparatively little is known about metabolic remodeling in the T1DM heart [2,16,17]. Thus, we focused on metabolic remodeling in the T1DM heart.

In the present study, we profiled cardiac metabolites and genes in adult, hyperglycemic, male Akita mice to identify key genes and metabolites indicative of disrupted metabolic pathways in the T1DM heart.

## 2. Results

### 2.1. Validation of Akita Mice

**Figure 1:**
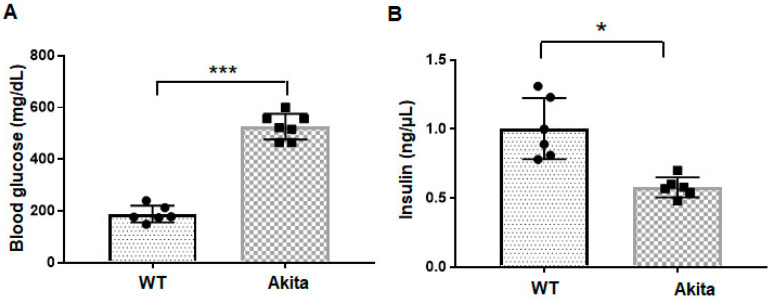
Validation of T1DM phenotype in the Akita mice. (**A**) Fasting blood glucose levels were measured by glucometer in WT and Akita mice. Values are mean ± SEM. *n* = 6. (**B**) Serum insulin levels were measured by ELISA method in WT and Akita mice. *n* = 5–6. Values are mean ± SE. Statistical analysis was conducted via a paired Student’s *t*-test. *** *p* < 0.001; * *p* < 0.05 (the exact *p*-value was 0.01).

Our earlier publications have reported the validation methods for T1DM in the Akita mouse [8,12]. We selected littermate WT from our Akita mouse breeding colony. We segregated Akita and WT mice based on the presence or absence of mutant *Ins2* band. To confirm the T1DM phenotype, we measured fasting blood glucose levels following our published protocol [8,12]. The blood glucose levels were higher in Akita mice (Figure 1A). We also measured serum insulin levels, which were decreased in the Akita mice due to the destruction of pancreatic beta cells (Figure 1B). Altogether, these features validate the T1DM phenotype of the Akita mouse.

### 2.2. Metabolomic Analyses

**Figure 2:**
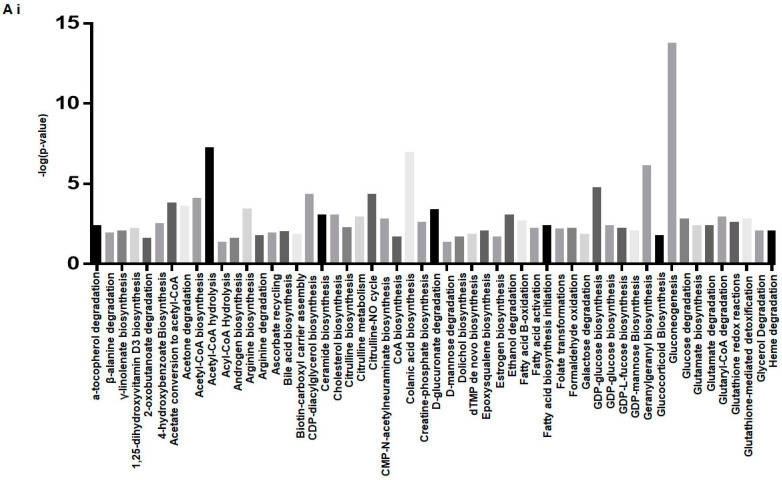

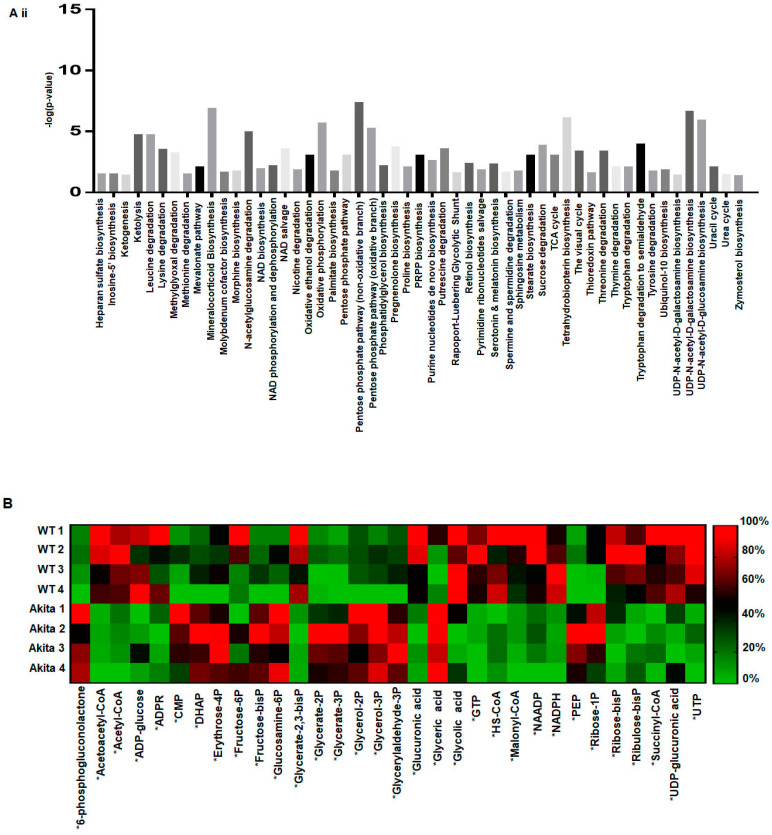
LC-MS metabolomics analysis of Akita compared to WT hearts. (**Ai**,**Aii**) Top predicted canonical metabolic pathways disrupted in the Akita heart with a –log(*p*-value) > 1.3 based on metabolite IPA analysis. (**B**) Heatmap indicating percent change of individual metabolites associated with the listed pathways in the Akita and WT hearts based on the expression log ratio calculated from individual metabolite expression; *p*-value < 0.05. *n* = 4.

**Figure 3:**
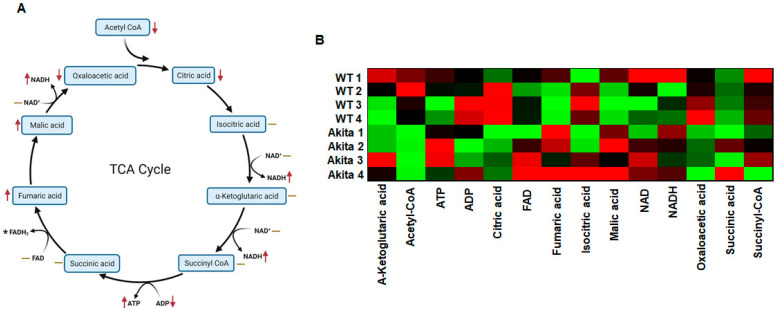
Differential expression of key metabolites involved in the tricarboxylic acid (TCA) cycle in the Akita heart by Ingenuity Pathway Analyses (IPA) of metabolites. (**A**) TCA cycle metabolite intermediates in Akita versus WT mice. Line indicates no change between groups. *FADH levels were undetected. (**B**) Heatmap indicating percent change of single metabolite levels associated with the TCA cycle in Akita and WT mice. Analysis conducted with Ingenuity Pathway Analysis software. *n* = 4.

For metabolomic analyses, we prepared LV tissue sample for performing LC-MS (Figure 2). The expression of all metabolites is shown in the Supplemental Data. We measured the expression of metabolites and performed Ingenuity Pathway Analysis (IPA) to predict the canonical metabolic pathways deranged in the Akita heart (Figure 2A). There were 108 metabolic pathways deranged in the Akita heart (Figure 2(Ai,Aii)). We presented percent change of individual metabolites associated with these disrupted metabolic pathways in the Akita heart (Figure 2B). Additionally, we performed IPA to analyze levels of tricarboxylic acid (TCA) cycle metabolites intermediates. We observed a downregulation in acetyl CoA, citric acid, oxaloacetic acid, and adenosine diphosphate (ADP) and an upregulation of NADH, fumaric acid, malic acid, and adenosine triphosphate (ATP) (Figure 3A). We also presented a heatmap showing the percent change of individual TCA metabolites between WT and Akita groups (red indicates high and green indicates low expression) (Figure 3B).

### 2.3. Genomic Analyses

**Figure 4:**
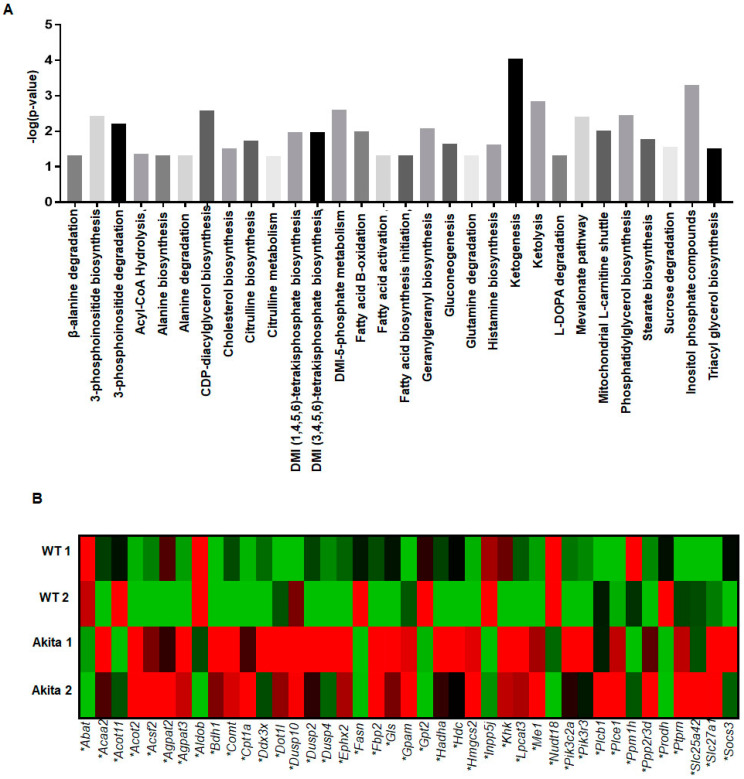
Genomics analysis of the Akita compared to WT hearts from next generation sequencing. (**A**) Based on Ingenuity Pathway Analysis (IPA) of genes obtained from next-generation sequencing showing the top predicted canonical metabolism pathways disrupted in the Akita heart with a –log(*p*-value) > 1.3. (**B**) Heatmap showing percent change in genes associated with the aforementioned metabolic pathways in the Akita heartbased on the expression log ratio calculated from FPKM values. All genes associated with the predicted pathways had a *p*-value < 0.05. *n* = 2.

To determine changes in gene expression, we isolated RNA from the LV tissue and performed next-generation sequencing. Total gene expression is presented in the Supplemental Data. Disrupted canonical metabolic pathways were based on individual gene expression in the Akita versus WT hearts using IPA (Figure 4A). There were 30 metabolic pathways deranged in the Akita heart (Figure 4A). The altered expression of key genes involved in these metabolic pathways are shown in the heatmap as percentage changed (red indicates high and green indicates low expression) of Figure 4B.

### 2.4. Genes and Metabolites Associated with the Disruption of Metabolic Pathways

**Figure 5:**
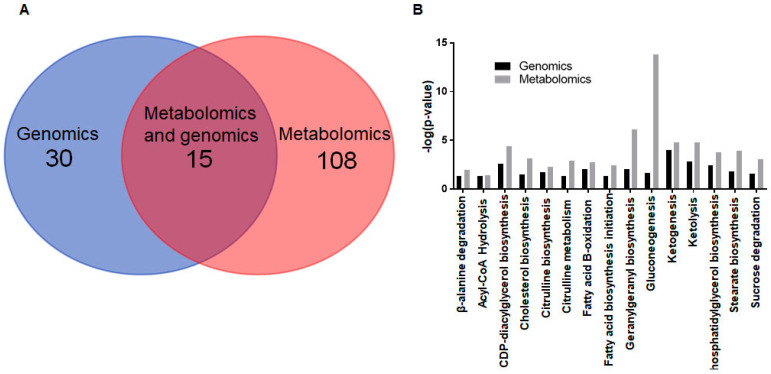
Independent and synergistic effects of genes and metabolites on metabolic remodeling in the Akita heart. (**A**) Overlap in genomic and metabolomic pathway analyses between disrupted metabolism pathways in the Akita heart. A total of 108 and 30 canonical metabolic pathways are predicted to be affected based on metabolomic and genomic analysis, respectively. There is an overlap of 15 disrupted metabolism pathways in the Akita heart between both analyses. (**B**) List of canonical metabolism pathways with a –log(*p*-value) > 1.3 commonly disrupted in both the genomic and metabolomic analysis in the Akita versus WT heart. Analysis was conducted with Ingenuity Pathway Analysis software. *n*= 2–4.

To determine the synergistic and independent effects of genes and metabolites behind T1DM-induced changes in metabolism, we selected the metabolic pathways that were disrupted in both the metabolomic and genomic datasets. Metabolomics indicated 108 disrupted metabolic pathways, and genomics indicated 30 disrupted metabolic pathways in the Akita heart (Figure 5A). We observed a total of 15 common deranged pathways from both genetic and metabolomic analyses (Figure 5B).

### 2.5. Upstream Regulators of Genes

We analyzed the genomic data to determine key transcription factors that serve as predicted upstream regulators of the 15 disrupted metabolic pathways from both the metabolomic and genomic datasets. These transcription factors are presented in Table 1.

## 3. Discussion

The prevalence of type 2 diabetes mellitus (T2DM) is higher (90–95%) when compared to T1DM (5–10%); thus, most cardiac metabolic studies are focused on T2DM. However, due to the heightened prevalence of diabetes, the number of total patients with T1DM is increasing rapidly. The severity of T1DM is relatively higher than T2DM due to increased fluctuations in blood glucose levels that lead to increased metabolic stress. However, there is a gap in knowledge as to which metabolic pathways are disrupted in the T1DM heart. Thus, investigating the underlying mechanisms behind the metabolic derangement in the T1DM heart is of high significance.

In the present study, we utilized next-generation RNA sequencing, LC-MS, and IPA to predict key disrupted metabolic pathways in the T1DM Akita heart. We revealed differentially expressed metabolites and genes and their disrupted metabolic pathways. We also predicted the transcription factors behind these differentially expressed genes in the Akita heart. Based on metabolomic and genomic data, we demonstrated novel synergistic and independent contributions of metabolites and genes in the metabolic derangement of the T1DM heart. Of note, disruption in ketogenesis, ketolysis, cholesterol biosynthesis, and fatty acid metabolism are demonstrated by comparing the metabolomic and genomic analyses, suggesting that these pathways have a critical role in metabolic remodeling in the T1DM heart. Thus, targeting these metabolic pathways could hold therapeutic potential in the amelioration of T1DM cardiomyopathy [18,19,20].

The heart has the highest demand of metabolic energy and the quickest rate of total ATP turnover. To meet this constant demand, metabolic flexibility allows the heart to utilize different substrates, including fatty acids (60–70%), glucose (20–30%), lactic acid, and ketone body for energy production [21,22]. Fatty acid has the highest oxygen/ATP ratio, making it the least efficient metabolic substrate, whereas ketone body has the lowest oxygen/ATP ratio, making it the mnost efficient metabolic substrate [23,24]. In the diabetic heart, fatty acid utilization is increased due to the lack of glucose uptake, following the substrate shift described by Randle’s glucose-fatty acid cycle [25]. This perturbations in metabolic substrate availability causes metabolic inflexibility in the diabetic heart, compromising glucose and ketone body utilization [26].

In T1DM patients, fatty acid utilization increases by 20%, while glucose utilization decreases by 50% in the heart. Furthermore, overall ATP generation efficiency decreases [27]. In the Akita heart, we have reported an increase in lipid droplet accumulation, demonstrating cardiac steatosis [12]. This is due to increased reliance on fatty acid metabolism, leading to the myocardial fatty acid deposition behind mitochondrial dysfunction, ROS generation, cell death, and cardiac remodeling [28,29,30]. While certain instances of intensive hyperglycemic treatment were found to increase the risk of DM-induced cardiac dysfunction, other medications used to manage diabetes prevent cardiac dysfunction [31,32]. Specifically, metformin and SGLT2 inhibitors decrease the risk of diabetes-induced cardiac dysfunction but have opposing effects on substrate metabolism [33,34]. Statins, medications used to prevent high cholesterol, also significantly lower the risk of DM-induced heart failure while directly inhibiting cholesterol synthesis [35]. GLP-1 agonists also decrease DM-induced cardiac dysfunction by increasing glucose and carbohydrate oxidation [36]. As the cardiometabolic effects behind medications commonly used to manage DM are defined, understanding which metabolism pathways are altered and their regulation in the DM heart holds increased therapeutic potential.

The metabolic status of the T1DM and T2DM heart differ greatly. Ketone body specifically plays a pivotal role in the metabolic remodeling of the T1DM heart [15,37]. While ketogenic diets improve cardiac efficiency in T2DM patients by increasing levels of circulating ketone body, they also lead to cardiovascular complications in T1DM [38,39]. Based on the shared disrupted metabolic pathways from our metabolomic and genomic analyses, we propose that ketogenesis, ketolysis, and fatty acid metabolism are key metabolic pathways pivotal for the cardiac metabolic derangement and could be targeted to improve energy efficiency in the T1DM heart.

Metabolic pathways are regulated both at the genomic and metabolomic level. Transcription factors such as p63, Sp1, Srebp-1c, Jun, and Creb1, affect a variety of intracellular mechanisms in addition to regulating glucose, fatty acid, and carbohydrate metabolism [40,41,42,43,44]. Other transcription factors, such as FoxO1, specifically target genes associated with fatty acid metabolism and gluconeogenesis [45]. Metabolites act as growth or starvation indicators, regulating metabolic pathways through negative feedback loops and direct protein interaction. Glycolytic intermediates, such as fructose-bisphosphate and glyceraldehyde 3-phosphate, upregulate glucose metabolism and downregulate lipogenesis [46,47]. Acetyl-CoA acts as an acetyl donor to induce fatty acid metabolism, while its carboxylated form, malonyl-CoA, inhibits fatty acid metabolism [48,49]. Nicotinamide adenine dinucleotide phosphate (NADPH) induces lipogenesis and carbohydrate metabolism via ADP-ribosylation; however, the addition of a nicotinic acid creates a potent inducer of Ca^2+^ release via nicotinic acid adenine dinucleotide phosphate (NAADP) [50,51]. Increased ketogenesis is observed in the T1DM heart and thus is associated with increased cardiovascular risk [15,52]. Thus, differential expression of these transcription factors has important roles in the upstream regulation of metabolic remodeling in the T1DM heart.

The male Akita mouse develops chronic hyperglycemia and hypoinsulemia similar to T1DM patients. In the Akita mouse, T1DM is induced by a G-to-A point mutation in the *Insulin 2* gene, resulting in a cysteine-to-tyrosine substitution that severs a disulfide bond and induces pro-insulin misfolding. The accumulation of misfolded pro-insulin leads to endoplasmic reticulum stress-induced pancreatic beta cell death, resulting in hypoinsulemia at four weeks of age [6,53]. Akita male mice survive 43 weeks, which is approximately half of the life span of normal mice [54]. Furthermore, they display signs of cardiac dysfunction 12 weeks of age, when their average blood glucose levels reach a minimum of 400 mg/dL. The Akita mouse exhibits cardiac fibrosis, hypertrophy, and dysfunction, all of which are present in T1DM cardiomyopathy [12,18,55]. Accumulation of lipid droplets is the initial pathological remodeling, as demonstrated by cardiac tissue isolated from diabetic patients post 5-weeks of heart transplantation [56]. In the Akita mouse heart, lipid accumulation, lipotoxicity, and diastolic dysfunction have been demonstrated [12,13]. Additionally, Akita mice show signs of metabolic remodeling, such as decreased glucose uptake, increased lipid uptake, and decreased oxidative capacity [12,57]. The metabolic remodeling in the diabetic heart causes diastolic followed by systolic dysfunction [58]. It is well established that the Akita mouse displays cardiac metabolic remodeling prior to the development of diastolic dysfunction [12,13,18,55]. This makes the Akita model an excellent candidate for high-throughput studies emphasizing disrupted cardiac metabolic pathways.

The best control for Akita mice is their WT littermates, which have a near similar genetic background with exception of a mutant *Insulin 2* gene. We bred *Ins2*^+/−^ Akita male mice with *Ins2*^+/+^ WT female mice to obtain Akita and WT siblings (littermates). The crossbreeding of heterozygous *Ins2* alleles (+ and −) from Akita mice and homozygous *Ins2* alleles (+ and +) from WT mice generates the following offspring: +/+, +/+, +/−, and +/−, where +/+ corresponds to WT, and +/− corresponds to Akita. As *Ins2^−/−^* mice develop robust hyperglycemia to the extent that they do not survive to the postnatal age, we do not obtain any full knockouts of *Ins2* from crossbreeding [59].

We used only male Akia mice in our studies, as female Akita mice have transient hyperglycemia at the time of puberty, which only lasts until sexual maturity. The blood glucose levels in sexually mature female Akita mice range from normal to moderately high, suggesting a protective role of estrogen and prolactin in the prevention of pancreatic beta cell death [6]. To investigate the specific effects of estrogen in the Akita mouse, Akita males should be treated with estrogen, and pancreatic beta cell function should be assessed. Additionally, androgen hormones present in males, and thus, may affect metabolites and gene expression. However, our genomic panel did not observe the presence of nearly all genes associated with androgen production. This includes 17α- hydroxylase (*Cyp 17*), 3β-hydroxysteroid dehydrogenase (*Hsd*), and 17 β-hydroxysteroid dehydrogenases (*Hsd 17b*), all of which were undetected in our RNA sequencing data. The only exception was *Hsb 17b11*, which showed a significantly higher expression in the Akita group.

While insulin is used as a frontline treatment in patients with T1DM, we did not treat our Akita mice with insulin. This allowed us to evaluate the full extent of metabolic remodeling caused by hyperglycemia without the variable of insulin-affected metabolic pathways in the T1DM heart. Although insulin treatment decreases blood glucose levels, improves antioxidant capability, and regulate cardiac metabolism, intensive glycemic control in diabetic patients did not demonstrate an impact on reducing cardiovascular event [3,60,61]. Furthermore, the RECORD trial showed nearly double the incidence rate of heart failure between more intensive and less intensive glycemic control [3]. Thus, we assumed that insulin treatment after development of adverse cardiac remodeling would display little cardiac protection, if any, against disrupted metabolic pathways preceding T1DM cardiomyopathy.

### Limitations

The first limitation of this study is the relatively small sample size (*n* = 2 for genomic and *n* = 4 for metabolomic analysis) used. The two reasons for this were (a) the high cost for genomic and metabolomic analysis and (b) we have previously published non-metabolic focused genomic analysis comparing WT and Akita hearts with a higher n number [18]. The second limitation was the lack of female mice used; thus, sex as biological variable was not addressed. Matured Akita females do not exhibit prominent diabetic phenotypes. Future studies in models of T1DM in which both males and females develop prominent hyperglycemia, such as streptozotocin-induced T1DM, could unravel details behind metabolic derangement in the female T1DM heart. The third limitation was the absence of validated metabolites and gene expression. We focused utilized a high throughput perspective of genes and metabolites to predict the disruption of metabolic pathways in the Akita heart. Future studies involving loss- and gain-of-function of genes and with substrate limitations/supply for metabolic pathways will validate these metabolic pathways. The fourth limitation was a lack of analysis utilizing human T1DM heart tissue. Although the Akita mouse is a good model for understanding human pathophysiology in the diabetic heart, prominent cardioprotective therapies in mice have failed to replicate in humans [62]. Thus, these genes, metabolites, and metabolic pathways should be examined in T1DM human cardiac tissue.

## 4. Materials and Methods

### 4.1. Animals

We procured WT (C57BL/6J, stock # 000664) and Ins2^+/−^Akita (stock # 003548) mice from The Jackson Laboratory (Bar Harbor, ME, USA). These mice were maintained and bred in the animal care facility of the University of Nebraska Medical Center (Omaha, NE, USA). They were provided with standard rodent chow and water ad libitum. We used the left ventricle (LV) heart tissue from 14–16-week-old male Akita and the age and gender matched littermates/siblings, normoglycemic Ins2^+/+^ WT mice. These studies were completed following the National Institutes of Health guidelines and approved by the Institutional Animal Care and Use (IACUC) committee of the University of Nebraska Medical Center (protocol 19-054-06-FC, 5 June 2019).

### 4.2. Genotyping

**Figure 6:**
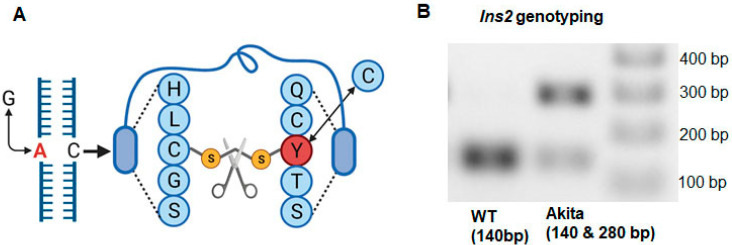
Characterization of the Akita mouse model. (**A**) Akita mice have a G-to-A point mutation in their *Insulin 2* (Ins2) gene, causing the severing of disulfide from a cysteine-to-tyrosine substitution. (**B**) Genotyping of Akita and littermate/sibling, normoglycemic WT mice. WT mice present a single band representing an allele at 140 base pairs (bp), while Akita mice demonstrate two bands at 140 and 280 bp, indicating the presence of two alleles.

We followed our recently published protocol of Akita genotyping [8]. In brief, DNA was isolated from a piece of tissue collected in a polymerase chain reaction (PCR) tube during ear punching. For DNA isolation, tissue was incubated in a lysis reagent (25 mM NaOH and 0.2 mM EDTA) at 98 °C for 1 h, hold at 4 °C, followed by addition of neutralizing reagent (40 mM Tris-HCl, pH 5.5) and brief centrifugation. The supernatant contained DNA. The concentration of DNA was measured by a nanodrop (# ND One-w, Thermo Fisher Scientific, Waltham, MA, USA). For PCR, 20 ng DNA was used for each PCR reaction. DreamTaq Green PCR Master Mix (#K1081, Thermo Fisher Scientific, Waltham, MA, USA) was used for PCR amplification. The PCR primers were Forward 5^/^TGC TGA TGC CCT GGC CTG CT 3^/^and reverse 5^/^TGG TCC CAC ATA TGC ACA TG 3^/^. The PCR reaction steps were: (1) 94 °C—3 min, (2) 94 °C—20 s, (3) 64 °C—30 s, (4) decrease temperature −0.5 °C per cycle, (5) 72 °C—35 s, (6) repeat step 2–5 for 12 cycles, (7) 94 °C—20 s, (8) 58 °C—30 s, (9) 72 °C—35 s, (10) repeat step 7–9 for 25 cycles, (11) 72 °C—2 min, and (12) hold at 4 °C for infinite time. The resulting amplified DNA underwent restriction digestion using SatI (#ER1642, Thermo Fisher Scientific, Waltham, MA, USA) before gel electrophoresis. For restriction digestion, the PCR product was restriction digested, where 4 µL of PCR product was mixed with 0.6 µL of Satl, 1 µL of 10× buffer G and the rest of the nuclease-free water for a 10 µL reaction. These components were mixed and spun down and incubated at 37 °C for at least 3 h and then stored at −20 °C. The product was used in agarose gel electrophoresis (2% agarose gel with 0.003% ethidium bromide) and visualized under ultraviolet light for DNA bands. The Akita mouse has mutation in one of *Insulin* 2 gene and it is heterozygous for *Insulin 2* gene (Ins2^+/−^) (Figure 6A). It shows both WT and mutant bands at 140 and 280 base pairs (bp), respectively (Figure 6B). The WT mouse shows only one band at 140 bp (Figure 6B).

### 4.3. Blood Glucose Measurement

A detailed protocol for blood glucose measurement in Akita mice was elaborated in our earlier publications [8,18]. In brief, mice were fasted for 6–8 h by removing food and allowing water ad libitum. At the time of blood glucose measurement, the tail was cleaned using 70% ethanol and pricked with a needle superficially close to the tip. Then. 5 µL of blood were collected on a glucose strip, which was inserted into an Accu-Chek Aviva glucometer (#05911958002, Roche, Indianapolis, IN, USA) for glucose measurement.

### 4.4. Serum Insulin Measurement

We measured serum insulin levels using an enzyme-linked immunoassay (ELISA) Ultra-Sensitive Mouse Insulin kit (#90080, Crystal Chem, Elk Groove Village, IL, USA,) following the manufacturer’s protocol. In brief, 400 μL of blood was collected from the vena cava and incubated at room temperature for 30 min before being centrifuged at 2000× *g* at 4 °C for 10 min. The top supernatant containing serum (approximately 200 µL) was collected. We mixed 10 µL of serum with 90 μL of dilutant supplied by the manufacturer, adding the dilution to the ELISA microplate well before incubating the plate at 4 °C for 2 h. After washing the plate with the wash solution supplied by the manufacturer, we added 100 µL of the conjugate and incubated the plate for 30 min at room temperature. We washed the plate, added 100 µL of substrate, and incubated for 40 min at room temperature. After washing the plate, we added 100 µL of stop solution to halt the resulting reaction. The plate was transferred to a plate reader (#E8032, Promega, Madison, WI, USA), and absorbance was measured at 450/630 nm.

### 4.5. Overall Study Design for Metabolomics and Genomics Analyses

**Figure 7:**
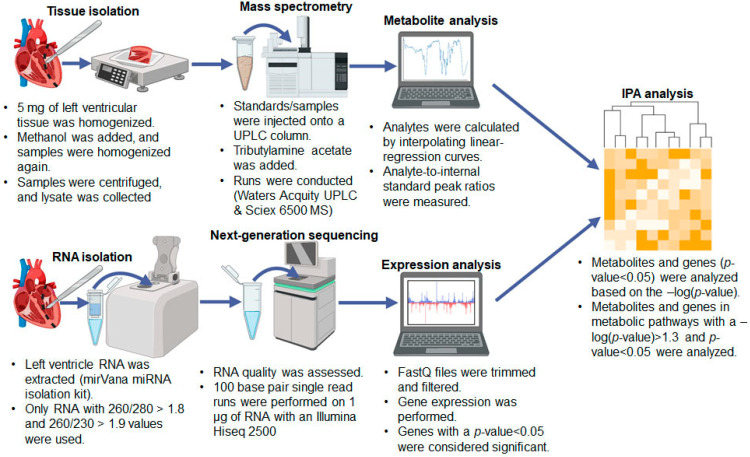
Overall study design for metabolomic and genomic analysis of heart tissue of Akita. Tissue was isolated specifically from the heart’s left ventricle (LV). LV tissue was prepared for liquid chromatography-mass spectrophotometry (LC-MS), and metabolites were analyzed. Ingenuity Pathway Analysis (IPA) was used to determine the association of metabolites with metabolic pathways. LV tissue was also used for RNA isolation and next-generation sequencing to evaluate gene expression. IPA was performed on differentially expressed genes to evaluate their roles in disrupted metabolic pathways. Both metabolomic and genomic analyses were compared to identify key pathways behind the regulation of metabolic remodeling in the Akita heart.

The overall study design is presented in Figure 7. After validating the T1DM phenotype, we used LV heart tissue for both metabolomic and genomic analyses. A previous publication from our laboratory described the genomic analysis of Akita and WT hearts [18]. We have also reported that an Akita mouse at the age of 14–16 weeks develops cardiac fibrosis, hypertrophy, lipid droplet accumulation, and cardiac dysfunction [12,18]. In the present study, we used an *n* = 4 for metabolomic analysis and an *n* = 2 for genomic analysis. The details of sample preparation and both metabolomic and genomic analyses are elaborated upon below.

### 4.6. Liquid Chromatography-Mass Spectrometry (LC-MS) Sample Preparation

To evaluate metabolites, we performed LC-MS, and 3-mm metal beads were added to 10 μL of water and 5 mg of left ventricular heart tissue. These were homogenized twice (30 Hz, 1 min each) with a MM 400 mill mixer. The process was repeated twice after adding 40 μL of methanol to the homogenate. The samples were sonicated in an ice-water bath for 3 min and centrifuged at 21,000× *g* and 5 °C for 10 min. The resulting supernatants were used for LC-MS analyses.

### 4.7. LC-MS Metabolite Analysis

For non-sugar metabolites, 10 μL of sample supernatant was mixed with 90 μL of a serially diluted standard solution (0.0002–10 μM) of each metabolite (NAD, NADH, AMP, GMP, UMP, UTP, ATP, GTP). Then, 10 μL of each sample/standard solution was injected onto a C18 UPLC column (2.1 × 100 mm, 2.5 μm) for UPLC-MRM/MS runs with (−) ion detection on a Acquity UPLC system (Waters, Milford, MA, USA) coupled to a 6500 Plus MS instrument (Sciex, Framingham, MA, USA), with the use of a tributylamine acetate-acetonitrile gradient elution buffer (5–50% acetonitrile, 22 min) at 0.25 mL/min and 50 °C.

For sugar metabolites, 20 μL of sample supernatant or serially diluted metabolite standards were mixed with 20 μL of a 13C6-glucose/13C6-glucose-6P standard solution, 40 μL of a 25 mM AEC solution, 20 μL of a 50 mM NaCBH3 solution, and 8 μL of LC/MS grade acetic acid, in turn. The resulting reaction from the mixtures took place at 60 °C for 70 min. Following this, 120 μL of water and 120 μL of chloroform were added. After vortex-mixing for 15 s and centrifugation for 5 min, 50 μL of the resulting supernatant was mixed with 450 μL of water. Then, 10 μL of this mixture was injected onto a PFP LC column (2.1 × 150 mm, 1.7 μm) for LC-MS on an 1290 UHPLC system (Agilent, Santa Clara, CA, USA) coupled with a 6495B Triple Quadrupole LC/MS system (Agilent, Santa Clara, CA, USA ) with positive-ion detection for quantification.

Metabolite-to-internal standard peak ratios were calculated based on internal standard calibration by interpolating the constructed linear-regression curves of individual compounds, resulting in quantifiable levels of individual metabolites. The LC-MS analysis was performed by Creative proteomics, New York, NY, USA.

### 4.8. RNA Extraction

RNA was extracted from LV tissue with a mirVana miRNA isolation kit (#AM1560, Thermo Fisher Scientific, Waltham, MA, USA) following the manufacturer’s protocol. In brief, 3 mg of tissue was homogenized in 300 μL of lysis/binding buffer supplied by the manufacturer and vortex-mixed for 30 s and incubated on ice for 10 min. Following this, 300 μL of Acid-Phenol: Chloroform was added to the homogenate, vortex-mixed for 60 s, and centrifuged at 10,000× *g* for 5 min at room temperature to allow phase separation. Without disturbing the interphase, supernatant (approx. 200 µL) was slowly pipetted into a fresh nuclease-free tube. We added 1.25-times volume (250 µL) of 100% ethanol to the supernatant and mixed gently by hand. The total volume was transferred to a filter column (maximum volume 700 µL per tube) and centrifuged at 10,000× *g* for 15 s. The column was washed three times (first by 700 µL of buffer 1 and two times with 500 µL of buffer 2/3) at 10,000× *g* for 8 s each. After removal of washing solution, the filter was centrifuged for 1 min at 10,000× *g* to remove all residual solution. The filter was transferred to a new tube, and 100 µL of elusion buffer heated to 95 °C was added to the middle of the column. The tube was then centrifuged at maximum speed (10,000× *g*) for 30 s, and the elute was collected. The quality and quantity of RNA were measured with a NanoDrop One (# ND-One-W, Thermo Fisher Scientific, Waltham, MA, USA). Only RNA with sufficient purity ratios (260/280 > 1.8 and 260/230 > 1.9) was used.

### 4.9. Next Generation RNA Sequencing

We used high-quality RNA assessed by NanoDrop and confirmed by Bioanalyzer for next-generation sequencing. Libraries were generated using 900 ng of total RNA from each sample and a TrueSeq V2 RNA-Seq kit (Illumina Inc, San Diego, CA, USA) following the recommended procedure. Libraries were multiplexed and sequenced on a NextSeq550 DNA Analyzer (Illumina, San Diego, CA, USA) to generate an approximately total of 20 million 50 bp single reads for each sample.

For quantification, FastQ files were trimmed with fqtrim, and reads that overlapped with Truseq adapters were filtered. The reads were aligned, and differential gene expression was performed with the Tuxedo pipeline. Genes with a *p*-value and q-value of less than 5% were taken to be significant. The genes were sorted according to the absolute value of the test_stat parameter as calculated by cuffdiff. For any given gene, the expression level value was averaged for both samples (A and B) of both groups, and each value was divided by this average expression value and then visualized. Next-generation RNA sequencing was conducted at the University of Nebraska Medical Center’s DNA Core Facility. The individual gene expression values for each sample as well as the cuffdiff results for the comparison analysis are available as Supplemental Data.

### 4.10. Ingenuity Pathway Analysis

Genes associated with metabolic pathways and metabolites from the comparison analysis were analyzed using the Ingenuity Pathway Analysis (IPA) tool (QIAGEN, Germantown, MD, USA), based on the absolute value of the test_stat parameter. Murine genes and metabolites demonstrating both direct and indirect interactions were selected and analyzed based on their log2 fold change values (Exp Log Ratio).

### 4.11. Statistical Methods

Values were presented as the mean with standard error of the mean (mean ± SEM). To compare the means of both groups, we conducted a paired Student’s *t*-test with assumed gaussian distribution, unless stated otherwise. For statistical analysis, we utilized GraphPad Prism (version 7.04) from GraphPad Software Inc., San Diego, CA, USA.

## 5. Conclusions

By conducting metabolomics and genomics analyses of adult male Akita and WT mice, we unraveled key differentially expressed metabolites and genes in the T1DM Akita heart. Further analysis with IPA revealed predicted metabolic pathways and transcription factors disrupted in the Akita heart. Interestingly, 15 of the predicted disrupted metabolic pathways were synergistically regulated by both genes and metabolites differentially expressed in the Akita heart. These metabolic pathways could be promising for investigating the molecular mechanisms behind metabolic remodeling in the T1DM heart.

## Figures and Tables

**Table 1:** Upstream regulators of metabolic pathways that are differentially regulated in the Akita heart.

Transcription Factor (Upstream Regulator)	Target Genes from Dataset	Z-Score	Exp Log Ratio	Overlap *p*-Value
Creb1	*Cpt1a, Fasn, Gls*	2.848	0.046	7.03 × 10^−13^
Ctnnb1	*Cpt1a, Fasn, Gpam, Hadha, Hmgcs2, Me1*	3.12	0.105	2.33 × 10^−11^
Ep300	*Comt, Cpt1A, Dusp4, Fasn, Hmgcs2, Slc27a1, Socs3*	2.606	0.132	3.6 × 10^−10^
Foxo1	*Aldob, Cpt1A, Fasn, Gpam*	4.127	−0.204	4.38 × 10^−16^
Jun	*Comt, Ephx2, Socs3, Gls*	3.086	−0.266	2.44 × 10^−21^
Sp1	*Fasn, Hadha, Hdc*	3.121	0.118	4.45 × 10^−11^
Tp63	*Dusp10, Fasn, KhK, Prodh, Pik3r3,*	2.024	0.566	2.32 × 10^−5^

## Data Availability

Not applicable.

## Supplementary Materials

The following supporting information can be downloaded at: https://www.mdpi.com/article/10.3390/ijms23031392/s1.
